# Impact of the Glycemic Control and Duration of Type 2 Diabetes on Vitamin D Level and Cardiovascular Disease Risk

**DOI:** 10.1155/2020/8431976

**Published:** 2020-02-19

**Authors:** Thuraya A. Alaidarous, Noura M. Alkahtani, Ghadeer S. Aljuraiban, Mahmoud M. A. Abulmeaty

**Affiliations:** ^1^Clinical Nutrition Department, King Faisal Specialist Hospital and Research Centre, Riyadh, Saudi Arabia; ^2^Department of Endocrinology, King Faisal Specialist Hospital and Research Centre, Riyadh, Saudi Arabia; ^3^Department of Community Health Sciences, College of Applied Medical Sciences, King Saud University, Riyadh, Saudi Arabia; ^4^Medical Physiology Department, Faculty of Medicine, Zagazig University, Zagazig, Egypt

## Abstract

**Background and Aims:**

To investigate the impact of glycemic control and T2D duration on vitamin D status and cardiovascular disease (CVD) risk among Saudi patients.

**Methods:**

This case-control study was conducted in King Faisal Specialist Hospital, Saudi Arabia. A total of 25 nondiabetic controls and 92 patients with confirmed T2D, aged 20–60 years, were included. Patients with T2D were divided into the following groups based on disease duration (newly diagnosed: ≈6 months and long duration: ≥5 years) and glycemic control based on their glycated hemoglobin (HbA_1C_) level with a threshold of ≤0.053 mol/mol: newly diagnosed controlled (NC, *n* = 25), newly diagnosed uncontrolled (NU, *n* = 25), newly diagnosed uncontrolled (NU, *n* = 25), newly diagnosed uncontrolled (NU, *n* = 25), newly diagnosed uncontrolled (NU,

**Results:**

Our study showed that T2D duration was an independent predictor of vitamin D deficiency. The longer disease duration, the lower odds of being vitamin D deficient (odds ratio (OR) = 0.05, 95% CI: 0.01–0.29, *p* < 0.05). No significant association was observed between vitamin D and HbA_1C_ levels. In the NU group, CVD risk scores were directly correlated with serum 25(OH)D (*r* = 0.53, *p* < 0.05). No significant association was observed between vitamin D and HbA_1C_ levels. In the NU group, CVD risk scores were directly correlated with serum 25(OH)D (*r* = 0.53, *p* < 0.05). No significant association was observed between vitamin D and HbA_1C_ levels. In the NU group, CVD risk scores were directly correlated with serum 25(OH)D (

**Conclusion:**

Duration of diabetes rather than glycemic control is associated with vitamin D deficiency. Glycemic uncontrol may augment vitamin D deficiency-associated CVD risk in both newly diagnosed and old patients with type 2 diabetes.

## 1. Introduction

Since 1921, the role of vitamin D in calcium homeostasis and bone health has been identified [1]. Vitamin D is composed of two bioequivalent forms: D_2_, which is found in vegetables and dietary supplements, and D_3_, which is synthesized in the skin through sun exposure and found in some oily fish and fortified foods. Once absorbed, vitamin D is metabolized in the liver to 25-hydroxyvitamin D (25(OH)D) and subsequently converted into 1,25-dihydroxyvitamin D (1,25(OH)_2_D_3_) [2]. Deficiency in vitamin D is a global concern; approximately one billion people in the world have low vitamin D levels [3]. In the United States, according to the National Health and Nutrition Examination Survey 2011–2012, about 40% of men and women were vitamin D deficient [4]. In Saudi Arabia, approximately 81% of the different population groups has vitamin D deficiency, and this condition is more prevalent in women compared to men [5].

Extensive research has shown adverse relations of vitamin D deficiency with cardiovascular disease (CVD), arterial hypertension, adiposity, and type 2 diabetes (T2D) [6–10]. Diabetes is a serious public health issue in Saudi Arabia, as approximately one out of four Saudis is diagnosed with diabetes [11]. Since Saudis have a high prevalence of T2D [11] and overweight [12], vitamin D deficiency may place them at an additional risk of CVD, making a strong case for early intervention [13].

Experimental studies found that (1,25(OH)_2_D_3_) stimulated pancreatic *β*-cell to secrete insulin [14]. Another proposed mechanism regarding the role of vitamin D in T2D is that since vitamin D deficiency causes an increase in inflammatory markers, insulin resistance may develop [15]. However, other intervention studies found no improvement in glycemic control with vitamin D supplementation [9, 16]. In the other hand, T2D itself might influence the level of vitamin D, and there is limited data regarding the impact of T2D duration and control on vitamin D status and CVD risk. Thus, we aimed to investigate the impact of glycemic control and duration of T2D on vitamin D status, and whether deficiency in vitamin D is related to CVD risk scores.

## 2. Methods

### 2.1. Participants and Recruitment

This case-control study included 92 patients with confirmed T2D and 25 healthy individuals (controls) aged 20–60 years. The study was conducted in King Faisal Specialist Hospital & Research Centre in Riyadh, Saudi Arabia. Patients diagnosed with type 1 diabetes, patients with history of endocrine disorders, patients with neurological disease, patients with renal disease and diabetic nephropathy, pregnant and lactating women, patients taking vitamin D supplementation within the last 6 months or any drugs affecting calcium and vitamin D metabolism like steroid medications, and patients taking antiepileptic medications and weight reduction drugs, which might affect the absorption of vitamin D, were excluded. Study protocol was approved by the Ethics Committee of the College of Applied Medical Sciences, King Saud University at all sites (reference no: CAMS 30/3536) and that of the King Faisal Specialist Hospital & Research Centre (KFSHRC) (Project no: 2151144).

Recruitment was done through screening of the medical history data from medical files based on the duration of T2D (newly diagnosed: ≈6 months and long duration: ≥5 years) and glycated hemoglobin (HbA_1C_) level with a threshold of ≤0.053 mol/mol. Patients were then approached and invited to participate. A total of 117 participants from various clinics at the endocrinology and family medicine units in King Faisal Specialist Hospital & Research Centre provided their written informed consent and were divided into five groups: the nondiabetic (N) control group (*n* = 25), the newly diagnosed controlled (NC) group (*n* = 25) (within 6 months of diabetes diagnosis and achieved glycemic goals based on levels of HbA_1C_ ≤ 0.053 mol/mol), the newly diagnosed uncontrolled (NU) group (*n* = 17) (within 6 months of diabetes diagnosis and were unable to achieve glycemic goals), the long duration controlled (LC) group (*n* = 25) (5–10 years of diabetes diagnosis and achieved glycemic goals of ≤0.053 mol/mol), and the long duration uncontrolled (LU) group (*n* = 25) (5–10 years of diabetes diagnosis and did not achieve their glycemic goals).

Sample size was calculated after considering the following: a 95% two-sided confidence interval and 80% power, and the case to control ratio was one to one. The hypothetical proportion of controls with vitamin D deficiency was about 51.7% [17], and that of cases with T2D approximately was 98.5% [18]. According to the OpenEpi software, version 2 [19], Fleiss formulae stated that the sample size of this case-control study is 13 cases, 13 controls, and the total sample size is 26 patients.

### 2.2. Medical History

On an assigned date, participants were requested to visit the clinics in the morning while still fasting (>12 hours); data on sex, age, occupation, region, marital status, smoking, eating habits, sun exposure, and other variables were collected using an interview-based health history questionnaire.

### 2.3. Biochemical Assessment

Blood (≈10 cc) was withdrawn after overnight fasting (>12 hours) and transferred immediately to a nonheparinizing tube for centrifugation. Fasting blood glucose (FBG) was measured using Roche/Hitachi modular Cobas c 701/702 [20]. Glycated hemoglobin (HbA_1C_) was measured using Cobas c 311, 501, and 502 analyzers, targeting an HbA1C level of ≤0.053 mol/mol (7.0%) [21]. All samples were measured in duplicates with intra- and interassay coefficient of variability (CV) of the total sample were about 5.1% and 14.7%, respectively. Vitamin D serum concentration and level of parathyroid hormone (PTH) were measured using electrochemiluminescence immunoassay, Cobas e411 autoanalyzer (Roche), Modular Analytics E170, and Cobas e 411, 601, and 602. The % of intra- and interassay CV of vitamin D duplicates for the total sample were 5.7% and 9.8%, respectively. For PTH, % of intra- and interassay CV of sample duplicates were 4.9% and 7.9%, respectively. Deficiency in vitamin D was defined as 25(OH)D < 50 nmol/l [1]. Markers of calcium homeostasis, alkaline phosphatase (ALP), and phosphorus were measured by Roche/Hitachi modular Cobas c 701/702 tests. Serum cholesterol, low-density lipoprotein (LDL), high-density lipoprotein (HDL), and triglycerides (TG) were assessed by Roche/Hitachi modular Cobas c 701/702 tests [20]. The % of intra- and interassay CVs were within the acceptable ranges.

### 2.4. Cardiovascular Disease Risk Scoring

We used three types of long-term cardiovascular disease (CVD) risk scores in this study. First was the Framingham 30-year risk score, calculated based on the lipid profile of patients with hard cardiovascular disease (FS30 lipid hard CVD). The second, FS30, used the lipid profile of patients with full CVD (FS30 lipid full CVD). The third score was calculated based on the patient's lifetime atherosclerotic CVD risk (lifetime ASCVD) and 10-year ASCVD risk, according to the American College of Cardiology/American Heart Association risk. A cutoff of ≤12% was used for FS30; therefore, participants with scores below the cutoff were classified as low risk. Scores of more than 12% were considered high risk. The lifetime ASCVD risk cutoff values were expressed in ASCVD percentage (5 and 8% for men and women, respectively) for participants aged 50 years with optimal risk factor levels. The 10-year ASCVD risk cutoff value was 0.6%. All of the scores included the following variables: age, sex, BMI, systolic blood pressure, lipids, smoking status, and diagnosis with diabetes or hypertension [22, 23].

### 2.5. Statistical Analysis

Statistical analyses were performed using the software package SPSS version 20 (SPSS, IBM, USA). All data were presented as means ± standard deviation (SD). Categorical variables were summarized using absolute and relative frequencies and compared using chi-square test. The respective subgroups and normal participants were compared using one-way analysis of variance with post hoc test. Pearson's correlation coefficients were used to test the correlation of diabetic and metabolic parameters with vitamin D. Multivariable logistic regression analysis was applied to identify the independent factors for the risk of vitamin D deficiency in patients with T2D. ORs and 95% confidence intervals (CI) for vitamin D deficiency were reported. A *p* value of ≤ 0.05 was considered significant.

## 3. Results

### 3.1. Descriptive Results

Of the total sample, 56.5% were women and 43.5% were men. The NC group showed more frequent family history of hypertension and cardiovascular disease in comparison to the control group (Table 1). The proportions of physically active participants in the control group and NC groups were 62.5% and 37.5%, respectively, in both sexes, while sun exposure was higher in the NC group compared to the normal group compared (*X*^2^ = 23.52, *p* < 0.01), and the time of exposure was usually at afternoon period. Additionally, the frequency of those with lifetime ASCVD risk (*p* < 0.05) and Framingham risk score (*p* < 0.01) was significantly higher in the NC group in comparison to the control group. The NU group (Table 2) showed more daily exposure to sun than normal group and usually afternoon. Regarding LC and LU groups (Tables 3 and 4), family history of hypertension and cardiovascular disease were more frequent in comparison to the control group, and the proportion of those who are exposed to the sun were more and usually at noon and afternoon periods.

### 3.2. Comparison of Study Variables between Case Groups and Control Group

Levels of FBG (*p* < 0.01) were significantly higher in the NU, LC, and LU groups compared with the control group (Table 5). Impact of disease duration was not significantly different when new cases were compared with long-lasting ones.

Paradoxically, LC group has significantly higher levels of 25(OH)D than normal population which was already VD deficient (*p* < 0.01). Moreover, PTH level was significantly lower in the LC in comparison to control (*p* < 0.05) (Table 5).

For CVD risk scores, the FS30 lipid hard CVD, FS30 lipid full CVD, and lifetime ASCVD were significantly different in the NC, NU, LC, and LU groups in comparison to control (Table 6). No significant changes in any risk were detected between controlled and uncontrolled groups or between new and long-lasting groups by post hoc test.

### 3.3. Correlation of 25-Hydroxyvitamin D Level with Cardiovascular Risk Scores among Study Groups

In the LU group, lifetime ASCVD risk showed significant negative correlation with serum 25(OH)D (*r* = −0.453, *p* < 0.05), while in the NU group, risk of Full Cardiovascular Disease scores was directly correlated with serum 25(OH)D (*r* = 0.525, *p* < 0.05) (Table 7).

### 3.4. Disease Duration of T2D and Glycemic Controllability as Determinant of Vitamin D Deficiency

Logistic regression analysis investigating the independent risk factors related to vitamin D deficiency in T2D patients showed that duration of T2D was associated with lower risk of vitamin D deficiency (defined by a cutoff 25(OH)D level below 50 nmol/l) (OR = 0.05, *p* < 0.05) (Table 8).

## 4. Discussion

Paradoxically in this case-control study, patients with longer disease duration had lower odds of being vitamin D deficient, and the mean of 25(OH)D was significantly higher in LC vs normal group. However, there was no significant association between vitamin D level and glycemic control based on FBG levels.

Interestingly, 72% of the control group had vitamin D deficiency, 20% had insufficient, and 8% had optimal levels of 25(OH)D. These findings regarding Saudi population are consistent with a recent published meta-analysis of all prevalence studies published between 2088 and 2015 [24]. Patients with T2D had a relatively low prevalence (48%) of low 25(OH)D level (<50 nmol/l). This is partly due to higher proportions of sun exposure in diabetic groups (Tables 1–4) and the usual follow up of this healthy lifestyle in addition to routine supplementation of vitamin D to T2D patients in the visited clinics in the KFSHRC.

Our findings also showed that the duration of T2D was an independent predictor of vitamin D deficiency. This result is compatible to findings of a recent cross-sectional study of an inverse association between disease duration and vitamin D [25]. Results from the landmark Women's Health Initiative Calcium/Vitamin D Trial have suggested that vitamin D oral supplements may delay the natural progression of T2D [26]. It was suggested that vitamin D supplementation of (2,000 IU/day for 16 weeks) improved pancreatic *β*-cell function in a randomized clinical trial, assessed using the disposition index [27]. In contrast, few cross-sectional studies found no significant association between the T2D duration and vitamin D deficiency [28, 29]. The differences observed between studies can be due to several factors. These include the nature of the study populations (ethnicity), lifestyle, medications administered, clinical characteristics of study participants, genetic variations in the vitamin D receptor, and PTH concentration.

We found no significant association between vitamin D deficiency and glycemic control (Table 7). These findings are comparable to results of a case-control study by Sheth et al., which indicated no significant association between deficiency of vitamin D and HbA_1C_ [30]. Similarly, another case-control study did not find a significant relation between HbA_1c_ and vitamin D levels in T2D patients [31]. Jorde et al. showed that supplementation with vitamin D (20000 IU every week for 5 years) in vitamin D-deficient persons did not halt progression from prediabetes to diabetes, suggesting an improbable association between blood glucose and vitamin D [32]. However, a recent cross-sectional study of 261 male and female participants aged 19 to 79 years found HbA_1C_ was inversely associated with 25(OH)D concentration [33]. Their findings were, however, based on a small population sample with a wide age range.

In the present study, FBG was significantly higher in both LC and LU subgroups than that in the control group. This finding may be indicative of insulin insensitivity [34]. Our findings are in agreement with a large Chinese cohort showing that the duration of diabetes increased the risk for insulin resistance [35]. Thus, FBG might be helpful in predicting the risk of diabetes in different ethnic populations [36].

In the current study, the full cardiovascular risk score in the NU group showed a significant direct correlation with serum 25(OH)D. On the contrary, there was an inverse correlation between 25(OH)D level and lifetime ASCVD risk score among the long duration uncontrolled diabetes group. These findings are consistent with those of a recent study that found an inverse correlation between 25(OH)D level and Framingham risk scores [37]. A population-based observational study investigated whether genetically low 25(OH)D levels were related to increased mortality and demonstrated increased all-cause mortality among vitamin D-deficient individuals [38]. There is no scientific evidence on the mechanism by which vitamin D confers a protective influence on the cardiovascular system; however, evidence suggests that vitamin D plays a role in the regulation of the renin-angiotensin system [38]. Furthermore, vitamin D plays a role in pathways that link inflammation and insulin resistance [39].

The current study has some limitations; these include the sample with significant differences in age between the controls and cases. Another limitation is that the observed paradox in the association between T2D and vitamin D deficiency may be due to the effect of statins. Previous research supports the effect of statins in conferring cardiovascular protection [40]. Thus, our findings need to be confirmed in larger prospective studies.

In conclusion, duration of diabetes is inversely associated with vitamin D level. Vitamin D plays a protective role on the cardiovascular system. Assessment of FBG level in addition to fasting blood glucose could contribute to the identification of more people with low vitamin D level. Glycemic uncontrol may augment vitamin D deficiency-associated risk of CVD in both newly diagnosed and old patients with type 2 diabetes.

## Figures and Tables

**Table 1:** Relevant medical and dietary variables among normal and newly diagnosed-controlled patients with T2D and their analysis by using chi-square test.

Variables	% within variable		Pearson chi-square	
	Normal	New & controlled	*X* ^2^	*p* value
*Gender*			0.33	0.564
Male	45.0	55.0		
Female	53.3	46.7		
*Employee*			16.09	0.001^∗^
No	6.7	93.3		
Yes	68.6	31.4		
*Family history of hypertension*			9.52	0.002^∗^
No	0.0	100.0		
Yes	59.5	40.5		
*Family history of CVD*			9.52	0.002^∗^
No	58.1	41.9		
Yes	0.0	100.0		
*Family history of diabetes*			6.818	0.009^∗^
No	0.0	100.0		
Yes	56.8	43.2		
*Past history of high blood pressure*			6.81	0.009^∗^
No	56.8	43.2		
Yes	0.0	100.0		
*Smoking status*			5.55	0.062
No	55.6	44.4		
Yes	0.0	100.0		
Passive	0.0	100.0		
*Physical activity*			5.55	0.018^∗^
No	27.8	72.2		
Yes	62.5	37.5		
*Bone symptoms*			12.50	0.001^∗^
No	62.5	37.5		
Yes	0.0	100.0		
*Exposure sun*			23.52	0.001^∗^
Weekly	73.5	26.5		
Daily	0.0	100.0		
*Time of exposure*			24.24	0.001^∗^
No	0.0	100.0		
Sunrise	83.3	16.7		
At noon	0.0	100.0		
Afternoon	0.0	100.0		
*Nature of work*			1.02	0.312
Inside	51.0	49.0		
Outside	0.0	100.0		
*Egg*			1.23	0.538
No	0.0	100.0		
Daily	42.9	57.1		
Weekly	52.4	47.6		
Monthly	—	—		
*Milk*			3.33	0.189
No	0.0	100.0		
Daily	42.1	57.9		
Weekly	58.6	41.4		
Monthly	—	—		
*Fish oil*			5.39	0.067
No	0.0	100.0		
Daily	—	—		
Weekly	41.7	58.3		
Monthly	58.8	41.2		
*Lifetime ASCVD risk*			8.14	0.004^∗^
No	100.0	0.0		
Yes	41.9	58.1		
*Framingham*			39.28	0.001^∗^
No	89.3	10.7		
Yes	0.0	100.0		

**Table 2:** Relevant medical and dietary variables among normal and newly diagnosed uncontrolled patients with T2D.

Variables	% within variable		Pearson chi-square	
	Normal	New & uncontrolled	*X* ^2^	*p* value
*Gender*			1.18	0.276
Male	50.0	50.0		
Female	66.7	33.3		
*Employee*			11.14	0.001^∗^
No	11.1	88.9		
Yes	72.7	27.3		
*Family history of hypertension*			10.29	0.001^∗^
No	0.0	100.0		
Yes	69.4	30.6		
*Family history of cardiovascular disease*			6.50	0.011^∗^
No	65.8	34.2		
Yes	0.0	100.0		
*Family history of diabetes*			3.08	0.079
No	0.0	100.0		
Yes	62.5	37.5		
*Past history of high blood pressure*			3.08	0.079
No	62.5	37.5		
Yes	0.0	100.0		
*Smoking status*			6.50	0.039^∗^
No	65.8	34.2		
Yes	0.0	100.0		
Passive	0.0	100.0		
*Physical activity*			3.46	0.063
No	38.5	61.5		
Yes	69.0	31.0		
*Bone symptoms*			6.50	0.011^∗^
No	65.8	34.2		
Yes	0.0	100.0		
*Exposure sun*			8.34	0.015^∗^
Weekly	67.6	32.4		
Daily	0.0	100.0		
*Time of exposure*			10.87	0.012^∗^
No	0.0	100.0		
Sunrise	62.5	37.5		
At noon	—	—		
Afternoon	0.0	100.0		
*Nature of work*			3.08	0.079
Inside	62.5	37.5		
Outside	0.0	100.0		
*Egg*			3.55	0.169
No	100.0	0.0		
Daily	57.9	42.1		
Weekly	—	—		
Monthly	0.0	100.0		
*Milk*			1.07	0.299
No	—	—		
Daily	72.7	27.3		
Weekly	54.8	45.2		
Monthly	—	—		
*Fish oil*			3.01	0.222
No	0.0	100.0		
Daily	—	—		
Weekly	45.5	54.5		
Monthly	66.7	33.3		
*Lifetime ASCVD risk*			5.71	0.017^∗^
No	100.0	0.0		
Yes	51.4	48.6		
*Framingham*			38.00	0.001^∗^
No	96.2	3.8		
Yes	0.0	100.0		

**Table 3:** Relevant medical and dietary variables among normal and long-lasting-controlled patients with T2D.

Variables	% within variable		Pearson chi-square	
	Normal	Long-lasting & controlled	*X* ^2^	*p* value
*Gender*			0.33	0.564
Male	45.0	55.0		
Female	53.3	46.7		
*Employee*			24.53	0.001^∗^
No	5.3	94.7		
Yes	77.4	22.6		
*Family history of hypertension*			6.81	0.009^∗^
No	0.0	100.0		
Yes	56.8	43.2		
*Family history of cardiovascular disease*			9.52	0.002^∗^
No	59.5	40.5		
Yes	0.0	100.0		
*Family history of diabetes*			3.19	0.074
No	0.0	100.0		
Yes	53.2	46.8		
*Past history of high blood pressure*			17.56	0.0011^∗^
No	67.6	32.4		
Yes	0.0	100.0		
*Smoking status*			6.81	0.033^∗^
No	56.8	43.2		
Yes	0.0	100.0		
Passive	0.0	100.0		
*Physical activity*			0.936	0.001^∗^
No	38.5	61.5		
Yes	54.1	45.9		
*Bone symptoms*			14.10	0.001^∗^
No	64.1	35.9		
Yes	0.0	100.0		
*Exposure sun*			17.56	0.001^∗^
Weekly	67.6	32.4		
Daily	0.0	100.0		
*Time of exposure*			19.90	0.001^∗^
No	0.0	100.0		
Sunrise	76.9	23.1		
At noon	0.0	100.0		
Afternoon	0.0	100.0		
*Nature of work*			1.02	0.312
Inside	51.0	49.0		
Outside	0.0	100.0		
*Egg*			5.09	0.165
No	0.0	100.0		
Daily	75.0	25.0		
Weekly	52.4	47.6		
Monthly	0.0	100.0		
*Milk*			9.29	0.026^∗^
No	0.0	100.0		
Daily	34.8	65.2		
Weekly	70.8	29.2		
Monthly	0.0	100.0		
*Fish oil*			4.75	0.093
No	0.0	100.0		
Daily	—	—		
Weekly	38.5	61.5		
Monthly	58.8	41.2		
*Lifetime ASCVD risk*			8.14	0.004^∗^
No	100.0	0.0		
Yes	41.9	58.1		
*Framingham*			46.15	0.001^∗^
No	96.2	3.8		
Yes	0.0	100.0		

**Table 4:** Relevant medical and dietary variables among normal and long-lasting-uncontrolled patients with T2D.

Variables	% within variable		Pearson chi-square	
	Normal	Long-lasting & uncontrolled	*X* ^2^	*p* value
*Gender*			0.333	0.564
Male	45.0	55.0		
Female	53.3	46.7		
*Employee*			8.00	0.005^∗^
No	10.0	90.0		
Yes	60.0	40.0		
*Family history of hypertension*			6.81	0.009^∗^
No	0.0	100.0		
Yes	56.8	43.2		
*Family history of cardiovascular disease*			12.50	0.001^∗^
No	62.5	37.5		
Yes	0.0	100.0		
*Family history of diabetes*			3.19	0.074
No	0.0	100.0		
Yes	53.2	46.8%		
*Past history high blood pressure*			12.50	0.001^∗^
No	62.5	37.5		
Yes	0.0	100.0		
*Smoking status*			12.50	0.002^∗^
No	62.5	37.5		
Yes	0.0	100.0		
Passive	0.0	100.0		
*Physical activity*			5.55	0.018^∗^
No	27.8	72.2		
Yes	62.5	37.5		
*Bone symptoms*			12.50	0.001^∗^
No	62.5	37.5		
Yes	0.0	100.0		
*Exposure sun*			28.12	0.001^∗^
Weekly	78.1	21.9		
Daily	0.0	100.0		
*Time of exposure*			24.03	0.001^∗^
No	0.0	100.0		
Sunrise	66.7	33.3		
At noon	0.0	100.0		
Afternoon	0.0	100.0		
*Nature of work*			1.02	0.312
Inside	51.0	49.0		
Outside	0.0	100.0		
*Egg*			2.36	0.307
No				
Daily	42.9	57.1		
Weekly	53.7	46.3		
Monthly	0.0	100.0		
*Milk*			4.08	0.130
No	0.0	100.0		
Daily	44.4	55.6		
Weekly	58.6	41.4		
Monthly				
*Fish oil*			7.14	0.028^∗^
No	0.0	100.0		
Daily	—	—		
Weekly	35.7	64.3		
Monthly	62.5	37.5		
*Lifetime ASCVD risk*			8.14	0.004^∗^
No	100.0	0.0		
Yes	41.9	58.1		
*Framingham*			42.59	0.001^∗^
No	92.6	7.4		
Yes	0.0	100.0		

**Table 5:** One-way ANOVA and post hoc test results of 25(OH)D and bone panel variable among study subgroups.

Groups	FBG (mmol/l)	HbA_1c_ (mol/mol)	25(OH)D (nmol/l)	Calcium (mmol/l)	PTH (ng/l)	PO4 (mmol/l)	ALP (U/l)
Normal	4.876 ± 0.61	0.052 ± 0.00	43.28 ± 17.96	2.316 ± 0.09	62.26 ± 26.40	1.10 ± 0.16	65.68 ± 16.20
NC	5.90 ± 0.90	0.060 ± 0.00	50.08 ± 18.12	2.29 ± 0.08	59.04 ± 24.59	1.04 ± 0.17	72.49 ± 15.81
NU	9.04 ± 2.08	0.087 ± 0.01	41.94 ± 22.35	2.32 ± 0.10	56.04 ± 11.80	1.16 ± 0.16	87.49 ± 24.81
LC	6.44 ± 0.94	0.064 ± 0.00	67.08 ± 26.42	2.35 ± 0.08	44.02 ± 11.20	1.18 ± 0.15	68.03 ± 13.55
LU	9.75 ± 2.56	0.094 ± 0.01	58.00 ± 26.78	2.31 ± 0.11	47.67 ± 15.54	1.12 ± 0.15	72.36 ± 16.25
*F* (between group)	41.484	102.172	4.973	1.184	3.898	2.879	4.657
*p* (value)	0.001^∗^	0.001^∗^	0.001^∗^	0.322	0.005^∗^	0.026^∗^	0.002^∗^
Post hoc test							
N vs. NC	0.148	0.007^∗^	0.826	0.969	0.977	0.575	0.626
N vs. NU	0.001^∗^	0.001^∗^	1.000	0.996	0.847	0.776	0.001^∗^
N vs. LC	0.005^∗^	0.001^∗^	0.003^∗^	0.592	0.011^∗^	0.469	0.989
N vs. LU	0.001^∗^	0.001^∗^	0.154	1.000	0.068	0.997	0.643
NC vs. NU	0.001^∗^	0.001^∗^	0.784	0.882	0.988	0.100	0.049^∗^
LC vs. LU	0.001^∗^	0.001^∗^	0.619	0.611	0.964	0.690	0.899
NC vs. LC	0.745	0.480	0.068	0.231	0.056	0.018^∗^	0.889
NU vs. LU	0.606	0.053	0.168	0.997	0.647	0.916	0.046^∗^

^∗^Note: data are presented as the mean ± SD. ∗*p* value for differences between groups according to ANOVA, significant if *p* < 0.05. 25(OH)D: 25-hydroxyvitamin D; PTH: parathyroid hormone; PO4: phosphorus; NC: newly diagnosed controlled; NU: newly diagnosed uncontrolled; LU: long-lasting diagnosed control; LU: long-lasting diagnosed uncontrolled.

**Table 6:** Cardiovascular disease risk among study groups vs. normal control.

Groups	Full CVD patient risk	Hard CVD patient risk	ASCVD risk 10 year	Lifetime ASCVD risk
Normal	7.56 ± 4.12	3.68 ± 2.26	0.00 ± 0.00	27.72 ± 13.58
NC	48.60 ± 19.00	36.12 ± 18.41	2.61 ± 1.85	51.80 ± 11.92
NU	48.53 ± 18.82	35.76 ± 17.88	4.00 ± 2.37	50.12 ± 10.27
LC	54.88 ± 19.14	39.56 ± 18.41	3.18 ± 2.23	55.40 ± 12.05
LU	52.88 ± 22.95	40.84 ± 20.32	2.44 ± 2.50	54.32 ± 9.81
*F* (between group)	29.837	21.33	2.418	23.888
*p* (value)	<0.0001	<0.0001	0.056	<0.0001
Post hoc test				
N vs. NC	<0.0001^∗^	<0.0001^∗^	0.323	<0.0001^∗^
N vs. NU	<0.0001^∗^	<0.0001^∗^	0.051^∗^	<0.0001^∗^
N vs. LC	<0.0001^∗^	<0.0001^∗^	0.149	<0.0001^∗^
N vs. LU	<0.0001^∗^	<0.0001^∗^	0.384	<0.0001^∗^
NC vs. NU	1.000	1.000	0.431	0.991
LC vs. LU	0.995	0.999	0.785	0.998
NC vs. LC	0.731	0.950	0.921	0.812
NU vs. LU	0.939	0.871	0.278	0.783

^∗^Note: data are presented as the *mean* ± *SD*. ∗*p* value for differences between groups according to ANOVA, significant if *p* < 0.05. Full cardiovascular disease: hard CVD or coronary insufficiency, angina pectoris, transient ischemic attack, intermittent claudication, or congestive heart failure. Hard cardiovascular disease: coronary death, myocardial infarction, or fatal or nonfatal stroke. ASCVD: atherosclerotic cardiovascular diseases defined as coronary heart disease death, nonfatal myocardial infraction, or fatal or nonfatal stroke.

**Table 7:** Correlation of 25-hydroxyvitamin D level with cardiovascular risk scores among different studied groups.

	Normal	Newly diagnosed controlled	Newly diagnosed uncontrolled	Long-lasting controlled	Long-lasting uncontrolled
ASCVD risk 10 year					
*r*	—	0.018	0.304	0.229	−0.109
Sig	—	0.940	0.337	0.306	0.603
Hard CVD patient risk					
*r*	−0.071	−0.036	0.446	−0.080	−0.124
Sig	0.735	0.864	0.073	0.705	0.555
Full CVD patient risk					
*r*	−0.027	0.048	0.525	−0.046	−0.150
Sig	0.898	0.820	0.031^∗^	0.828	0.475
Lifetime ASCVD risk					
*r*	0.074	0.220	0.012	−0.023	−0.453
Sig.	0.727	0.290	0.964	0.915	0.023^∗^
Fasting blood glucose level					
*r*	0.102	0.002	0.132	−0.111	0.032
Sig.	0.626	0.992	0.614	0.596	0.879

**Table 8:** Multiple logistic regression analysis of disease duration of T2DM and controllability HBA_1C_ as determinant of vitamin D deficiency.

	Vitamin D deficiency		*p* value
	Odd ratio	95% CI	
*Disease duration*	0.05	0.01–0.29	0.001^∗^
*Glycemic control*	0.27	0.07–1.06	0.061

^∗^Note: regression test *p* value 0.05. ^∗^CI: confidence interval 95 percentage.

## Data Availability

The data used to support the findings of this study are available from the corresponding author upon request.

## Abbreviations

1,25(OH)_2_D_3_: 1,25-Dihydroxyvitamin D3
25(OH)D: 25-Hydroxyvitamin D
ASCVD: Atherosclerotic cardiovascular disease
BMI: Body mass index
CVD: Cardiovascular diseases
FBG: Fasting blood glucose
FRS: Framingham risk score
HbA_1C_: Glycated hemoglobin
HDL: High-density lipoprotein
LDL: Low-density lipoprotein
T2D: Type 2 diabetes.
